# Binding and Regulation of Transcription by Yeast Ste12 Variants To Drive Mating and Invasion Phenotypes

**DOI:** 10.1534/genetics.119.302929

**Published:** 2019-11-25

**Authors:** Wei Zhou, Michael W. Dorrity, Kerry L. Bubb, Christine Queitsch, Stanley Fields

**Affiliations:** * Department of Genome Sciences, University of Washington, Seattle, Washington 98195; † Molecular and Cellular Biology Program, University of Washington, Seattle, Washington 98195; ‡ Department of Medicine, University of Washington, Seattle, Washington 98195

**Keywords:** gene expression regulation, genome binding, phenotype, transcription factor, transcriptome

## Abstract

Amino acid substitutions are commonly found in human transcription factors, yet the functional consequences of much of this variation remain unknown, even in well-characterized DNA-binding domains. Here, we examine how six single-amino acid variants in the DNA-binding domain of Ste12—a yeast transcription factor regulating mating and invasion—alter Ste12 genome binding, motif recognition, and gene expression to yield markedly different phenotypes. Using a combination of the “calling-card” method, RNA sequencing, and HT-SELEX (high throughput systematic evolution of ligands by exponential enrichment), we find that variants with dissimilar binding and expression profiles can converge onto similar cellular behaviors. Mating-defective variants led to decreased expression of distinct subsets of genes necessary for mating. Hyper-invasive variants also decreased expression of subsets of genes involved in mating, but increased the expression of other subsets of genes associated with the cellular response to osmotic stress. While single-amino acid changes in the coding region of this transcription factor result in complex regulatory reconfiguration, the major phenotypic consequences for the cell appear to depend on changes in the expression of a small number of genes with related functions.

## Abstract (precis)

Here, Zhou *et al.* took advantage of *Saccharomyces cerevisiae* and its well-characterized mating and invasion pathways to explain how transcription factor variants alter motif recognition and gene expression, and ultimately, organismal phenotypes...

THE binding of transcription factors to *cis*-regulatory sequence motifs controls the expression of target genes. Genetic variation in either a transcription factor or the *cis*-regulatory sequences to which it binds creates variation in gene expression, driving phenotypic variation, evolution, and disease. Emphasis has focused on *cis*-regulatory variation and its effect on gene expression for multiple reasons. First, regulatory variation likely played a large role in evolution because it resulted in less-severe pleiotropic effects on whole-organism phenotypes, allowing for subtle cell or tissue changes rather than interfering with overall body plans (Wittkopp and Kalay 2012). Second, many trait-associated variants in human genome-wide association studies reside in or near regulatory DNA (Maurano *et al.* 2012), with single-nucleotide changes in a transcription factor-binding site capable of altering factor occupancy and resultant gene expression (Borneman *et al.* 2007; Reddy *et al.* 2012; Deplancke *et al.* 2016). Third, high-throughput *in vitro* assays for examining the binding of transcription factors to DNA sequences, using protein-binding microarrays (Berger *et al.* 2006) or sequencing-based technologies (Jolma *et al.* 2010; Stormo and Zhao 2010; Slattery *et al.* 2011; Hughes *et al.* 2018), are more amenable to testing large numbers of DNA sequences than large numbers of transcription factor variants.

However, recent findings have highlighted the need to evaluate the functional consequences of transcription factor coding variants. Despite their high conservation overall, the coding sequences of human transcription factors show abundant genetic variation, suggesting that most individuals contain unique repertoires of variant factors (Barrera *et al.* 2016). Compared to individual regulatory sequence variants, individual transcription factor variants can exert a far larger phenotypic impact by changing the expression of multiple downstream target genes. Variation in the coding sequence of a transcription factor may change its DNA-binding affinity, specificity, or both by altering the factor’s structure, or its oligomerization with itself or with cofactors. Although *in vitro* studies and modeling can predict the effects of variation on transcription factor–DNA binding (Barrera *et al.* 2016), few *in vivo* studies connect altered transcription factor binding to altered gene expression and whole-organism phenotypes.

Here, we took advantage of *Saccharomyces cerevisiae* and its well-characterized mating and invasion pathways, in an effort to explain how transcription factor variants alter motif recognition and gene expression, and ultimately organismal phenotypes. Yeast mating and invasion pathways converge on the highly conserved fungal transcription factor Ste12, which interacts differentially with cofactors to activate either mating or invasion. In yeast mating, Ste12 binds at mating pheromone-responsive genes as a homodimer (Wong Sak Hoi and Dumas 2010), or with the cofactors Mcm1 (Mead *et al.* 2002) and Matα1 (Yuan *et al.* 1993); Mcm1 also binds to numerous other genes, including ones involved in cell cycle progression, cell wall and membrane synthesis, and metabolism (Kuo and Grayhack 1994). The Ste12 DNA-binding site is the pheromone response element, whose consensus sequence is TGAAACA. In invasion, which is triggered by increased temperature and lack of nutrients, Ste12 and its cofactor Tec1 are both required to activate target genes, some of which contain a Ste12-binding site near the Tec1 consensus sequence (GAATGT), forming the filamentation response element (Bardwell *et al.* 1998).

We have previously shown that single-amino acid changes in a region of the Ste12 DNA-binding domain can shift the trait preference of yeast cells toward either mating or invasion, and that, at least in some cases, these changes result in altered *in vitro* DNA-binding preferences of the Ste12 variant (Dorrity *et al.* 2018). To interrogate how these *in vitro* preferences translate into markedly different organismal phenotypes, we generated *in vivo* genome-binding profiles and expression profiles of wild-type Ste12 and six Ste12 variants, previously shown to alter mating or invasion phenotypes. We used the calling-card method (Wang *et al.* 2007) to identify genomic sites bound *in vivo* for each variant. Although each variant binds a set of genomic sites with high reproducibility, there is a comparatively low degree of overlap between the genome-wide profiles of Ste12 variants, even among variants with shared mating and invasion phenotypes. Nevertheless, we find examples of specific changes in binding sites and expression that likely contribute to the observed phenotypes. By integrating binding and expression data, we conclude that while subtle changes in the coding region of this transcription factor can result in a large reconfiguration of expression, the major determinants of organismal phenotypes are the changes in the expression of a small, related set of genes.

## Materials and Methods

### Construction of a deleted *STE12* strain

We generated yeast strains in the BY4705 *MAT*α background whose copy of the *STE12* gene was deleted by site-specific genomic deletion (Gray *et al.* 2005). In the first step, yeast was transformed with a PCR fragment that contained the *URA3* gene flanked at each end with 40-bp of sequence corresponding to the flanking sequences of the *STE12* ORF. The *STE12* ORF was replaced with *URA3* by selecting for transformants in Ura− medium. In the second step, a PCR fragment was amplified that contained sequence flanking both sides of the deleted ORF, and this fragment was transformed into the strain generated in step 1 by selecting for loss of *URA3* on 5-fluoroorotic acid (5-FOA) medium. While *STE12* deletion resulted in completely sterile strains that were complemented by a plasmid-borne wild-type *STE12* gene, we did not confirm that the *URA3* gene was fully deleted. Thus, in the following Ste12 variant RNA sequencing (RNA-seq), we carried out differentially expressed gene analysis after filtering out *URA3*.

### *STE12* variant RNA-seq

The *STE12* locus from *S. cerevisiae* strain BY4705, including the intergenic regions, was introduced into the yeast vector pRS415 containing a *LEU2* marker (Mumberg *et al.* 1995). Individual point mutations were generated in wild-type *STE12* plasmids by site-directed mutagenesis (Q5; New England Biolabs, Beverly, MA). Plasmids were transformed into yeast (BY4705 *MAT*α) with a deleted endogenous copy of *STE12* by high-efficiency lithium acetate transformation (Gietz and Woods 2002). Cells were grown to exponential phase (five replicates). RNA was extracted using acid phenol extraction, as previously described (Cuperus *et al.* 2015). Total complementary DNA (cDNA) was generated using anchored oligo-dT primer and SuperScript IV (Life Technologies) (Cao *et al.* 2017). Second-strand synthesis was carried out at 16° for 180 min with NEBNext Second Strand Synthesis module (New England Biolabs). cDNA was tagmented with a Nextera tagmentation kit (Illumina) at 55° for 5 min. The reaction was stopped by adding 1 × DNA binding buffer (Zymo Research) and incubating at room temperature for 5 min. Each well was then purified using 1.5 × AMPure XP beads (Beckman, Fullerton, CA) and eluted in 16 μl of buffer EB (QIAGEN, Valencia, CA). Each sample was then mixed with 2 μl of 10-μM indexed P5 and P7 primers, and 20 μl NEBNext High-Fidelity 2 × PCR Master Mix (New England Biolabs). Amplification was carried out using the following program: 72° for 5 min; 98° for 30 sec; 10 cycles of 98° for 10 sec, 66° for 30 sec, and 72° for 1 min; and a final 72° for 5 min. The library was purified with 0.8 ×AMPure XP beads (Beckman) and prepared for sequencing using Illumina NextSeq.

### Construction of plasmids for a PiggyBac-based transposon-based calling-card method

A donor plasmid carrying the PiggyBac transposon and an SP1-PBase helper plasmid were obtained from Robi Mitra (Washington University in St. Louis). To use G418 and 5-FOA selection in yeast, a *KanMX* gene was inserted into the transposon region. We added an 8-bp (NNNNNNNN) unique molecular identifier (UMI) sequence to the transposon region of each copy of the donor plasmid to quantify unique insertion events per cell. For the helper plasmid, we replaced SP1 with a wild-type and variant full-length *STE12* fragment and fused it with the PiggyBac transposase to encode Ste12-PBase, whose expression is under the control of the galactose-inducible *GAL1* promoter. Gibson assembly products were introduced into *Escherichia coli* and the plasmid was isolated. Constructs were confirmed by Sanger sequencing.

### Transformation of cells and transposition of PiggyBac

Plasmids used for transformation were prepared using a plasmid miniprep kit (QIAGEN) following the manufacturer’s protocol. Paired donor plasmid and helper plasmid were transformed into yeast (BY4705 *MAT*α) by high-efficiency lithium acetate transformation (Gietz and Woods 2002). After transformation, cells were collected and put on the induction plates with galactose. Cells were induced for 5 days to express Ste12-PBase. Cells were then collected, diluted back to OD_600_ = 0.45, and cultured in rich medium for 6 hr recovery (to OD_600_ = ∼1.6). Cells were put onto selection plates with 5-FOA and G418 at varying dilutions for ∼2–3 days.

### Inverse PCR

Cells were collected from selection plates and genomic DNA was extracted from each sample using the Smash and Grab method, as described by Hoffman and Winston (1987). Each DNA sample was divided into two 20-μg aliquots and digested by *Taq*I and *Rsa*I (New England Biolabs) individually. Digested products were purified by DNA purification column (Zymo Research) and ligated overnight at 15° in dilute solution to encourage self-ligation. Self-ligated DNA was purified by DNA purification column (Zymo Research) and used as the template in an inverse PCR. Primers that anneal to the PiggyBac donor sequences (primer 316: 5′-GATGTCCTAAATGCACAGCGAC-3′ and primer 317: 5′-GAGGCGTGCTTGTCAATGC-3′) were used to amplify the genomic regions flanking the transposon, and then adaptor sequences that allow the PCR products to be sequenced on Illumina sequencing platforms were added by primer 367 (5′-AATGATACGGCGACCACCGAGATCTACACCTCCATCGAGACACTCTTTCCCTACACGACGCTCTTCCGATCTCGTCAATTTTACGCAGACTATC-3′) and 377 (5′-CAAGCAGAAGACGGCATACGAGATTGCTTGTCAATGCGGTAAG-3′). The PCR products were purified using a DNA purification column (Zymo Research). For each sample, the same amount of PCR product from digestion with each restriction endonuclease was pooled and submitted for Illumina sequencing.

### Processing of PiggyBac transposon-based calling-card data

Sequencing was completed on Illumina’s NextSeq platforms. The raw data included all fastq files from the PiggyBac transposase-only control, wild-type Ste12, and the six variants. Each sample had two replicates. In read 1, the first segment was the universal primer sequence—5′-CGTCAATTTTACGCAGACTATCTTTCTAGGG-3′—followed by the flanking genome sequence of 39 bp. The first 8 bp of read 2 was the UMI sequence used to identify unique insertion events. We first filtered sequence reads with high quality and mapped them back to the yeast genome. Then we quantified independent PiggyBac insertions based on the UMIs detected in each sample. Finally, we called significant PiggyBac insertion peaks by MACS2, a peak-calling algorithm (Zhang *et al.* 2008). Target genes were then assigned to insertion peaks that were within 1000-bp 5′ or 200-bp 3′ of the transcription start site for that gene (Wang *et al.* 2011). For each gene, we normalized the detected UMIs to the total UMIs in each sample, log-transformed the data, and generated a gene count matrix. We determined the fraction of total UMIs near a subset of genes and then performed a proportion test to identify genes with a significantly changed fraction of UMIs mapping in the Ste12 variant compared to in the wild-type.

### Motif analysis for PiggyBac transposon-based calling-card data

Insertions with counts above the 85th percentile were identified as “high-count insertions.” We identified 300-bp windows around each high-count insertion, and then merged the windows (bedops -m) to generate high-insertion-count sites for each replicate. We extracted the sequence from these windows and attempted to identify *de novo* motifs using MEME (Bailey *et al.* 2009). We also scanned these sequences for a set of known yeast motifs (Teixeira *et al.* 2014) using fimo (Grant *et al.* 2011). We tallied the coincidence of known motifs, normalizing by the number of merged high-insertion-count windows. We used DESeq2 (Love *et al.* 2014) to identify motif pairs that appeared at different frequencies in the variants, taking advantage of the replicate data. We identified 529 pairs with log10 (mean motif-pair count across transcription factor variants) > 1 and dispersion levels greater than expected for that mean.

### HT-SELEX

We purified fragments of Ste12(1–215) expressed from pGEX-4T-2 vectors. These protein fragments have been used previously (Dorrity *et al.* 2018) and are sufficient for binding *in vitro*. Fragments of wild-type and variant Ste12 proteins were purified using a GST tag, and used for HT-SELEX (high throughput systematic evolution of ligands by exponential enrichment). SELEX reactions with homogenous and mixed protein populations were performed identically to previous work (Jolma *et al.* 2015). Briefly, a 50-μl reaction containing purified Ste12 (1:25 M ratio with DNA), 200 ng nonspecific competitor double-stranded nucleic acid poly (dI/dC), and 100 ng selection ligand (36N) were incubated in binding buffer [140 mM KCl, 5 mM NaCl, 1 mM K_2_HPO_4_, 2 mM MgSO_4_, 20 mM HEPES (pH 7.05), 100 μM EGTA, and 1 μM ZnSO_4_] for 2 hr. GST Sepharose (GE Healthcare) beads were added to each reaction, incubated for 30 min, and unbound ligand was removed using seven buffer washes. Output reactions were amplified by PCR after each round and these products were subsequently used to prepare high-throughput sequencing libraries. SELEX motif enrichments were analyzed using Autoseed software (Jolma *et al.* 2015). The pool of binding-selected output sequences was compared against a fully random input sequence pool to identify enriched motif sequences.

### Motif analysis for HT-SELEX data

All possible 10mers were computed among the bound sequences observed in round five of SELEX for each Ste12 variant, a pool that should contain an enrichment of bound sequences. The 10mer counts for all output sequences were then normalized to the counts of each of those 10mers in the random oligo pool used as the input ligand for HT-SELEX. For each variant, the top enriched sequences were determined as those whose 10mer enrichment was three SD above the mean enrichment of all 10mers. WebLogos were generated using MEME by searching for enriched motifs among these highly enriched 10mer sequences for each variant.

### Data availability

High-throughput sequencing reads, along with data sets showing calculated insertion scores for each variant (two replicates) and transcriptome data (five replicates), have been submitted to the Gene Expression Omnibus (GEO) under accession numbers: GSE141713. Supplemental material available at figshare: https://doi.org/10.25386/genetics.10326800.

## Results

### A PiggyBac-based calling card identifies binding sites of Ste12 and Ste12 variants

**Figure 1:**
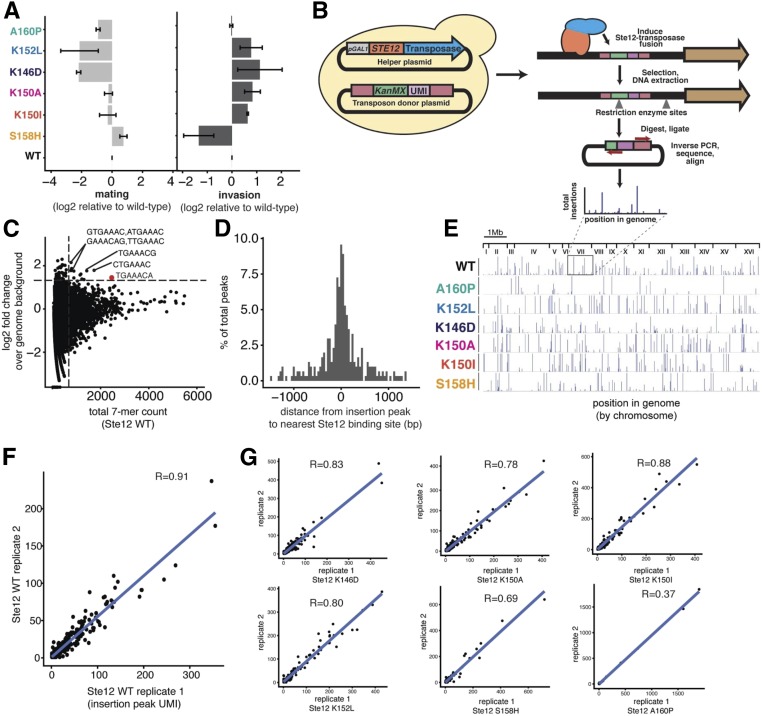
A PiggyBac transposon-based calling-card method identifies genome-wide binding profiles for WT Ste12 and its six variants. (A) Mating and invasion phenotypes of selected Ste12 variants (Dorrity *et al.* 2018); the *x*-axis shows the mating or invasion score based on high-throughput trait selection assay, the *y*-axis shows six variants, error bars indicate SEs of replicates, and variants with different phenotypes are annotated by different colors. (B) PiggyBac transposon-based calling-card method workflow with key steps. (C) 7-mer analysis of the region 100 bp around each insertion peak. Each point represents a unique 7-mer sequence; the *x*-axis shows the total count of each 7-mer and the *y*-axis shows the relative enrichment of each 7-mer over the genome background. Dashed lines represent the count threshold used to identify enriched 7-mers. (D) The distribution of distances between each insertion peak and its closest Ste12-binding site. The *x*-axis specifies the distance from the center of the Ste12-binding site. The *y*-axis is the proportion of detected insertion peaks. (E) The genome-wide PiggyBac insertion patterns of each variant shown along the 16 *S. cerevisiae* chromosomes. (F) Correlation analysis between two WT Ste12 replicates in the PiggyBac transposon-based calling-card experiments. (G) Correlation analysis between the two replicates for each of the six Ste12 variants in the calling-card experiments. UMI, unique molecular identifier; WT, wild-type.

We previously conducted a deep mutational scan of a segment of the Ste12 DNA-binding domain, and subjected yeast cells carrying a library of these variant Ste12 proteins to selection for either mating or invasion (Dorrity *et al.* 2018). For the mating selection, *MAT***a** cells with *STE12* variants were mixed with *MAT*α cells and selected using auxotrophic markers that would be present only in mated diploids. For the invasion selection, the yeast were incubated on plates until invasion had occurred and then washed from the plate surface, such that only cells embedded in the agar should remain. We identified Ste12 variants with single-amino acid changes in the DNA-binding domain that altered the preferences of yeast cells in their mating or invasion traits. To determine how the genomic targets of Ste12 might differ depending on these amino acid changes, we chose six Ste12 variants with altered mating or invasion phenotypes (Figure 1A, variants annotated by color). We included three variants that caused reduced mating, one of which led to wild-type invasion (A160P) and two to hyper-invasiveness (K152L and K146D). We included three other variants that mate like wild-type yeast, two of which are hyper-invasive (K150A and K150I) and one defective for invasion (S158H).

We used the transposon-based calling-card method (Wang *et al.* 2007) to identify the binding sites of these variants *in vivo* and genome-wide. This method fuses a transcription factor to a transposase such that the transcription factor directs transposon insertion into the genome at a TTAA site nearby to where it is bound. Following the transposition events, genomic sites that were used for transposition were amplified and characterized by high-throughput DNA sequencing (Wang *et al.* 2011). We designed two plasmids for use of this method in yeast with the PiggyBac transposon (Figure 1B). First, we constructed a donor plasmid that carries the PiggyBac transposon containing a G418-resistance marker (*KanMX*) and a *URA3* marker. We quantified unique insertion events per cell using a random 8-bp UMI sequence in the transposon region of each copy of the donor plasmid. Second, we constructed a helper plasmid that encodes a fusion of the full-length Ste12 protein to the PiggyBac transposase (Ste12-PBase), whose expression is under the control of the galactose-inducible *GAL1* promoter. We cotransformed both of these plasmids into the *MAT*α *STE^+^* BY4705 strain (Brachmann *et al.* 1998). This mating-competent strain was used because the Ste12-PBase acts dominantly in transposition and would be induced in cells that have an intact mating regulatory program.

We induced the Ste12-PBase, which results in the insertion of the transposon near sites bound by a variant Ste12 protein and the conversion of cells with these insertions to G418 resistance. We then measured the chromosomal acquisition of the transposon-borne *KanMX* marker in cells that had lost the donor plasmid. The cells were identified by selection in media with G418, which requires the *KanMX* marker, and with 5-FOA, which is toxic for cells that have Ura3 activity (Boeke *et al.* 1987). We found that the transposition efficiency of PiggyBac in yeast is ∼10% (Supplemental Material, Figure S1). To reveal the genomic DNA sequences flanking the end of the transposon, we isolated genomic DNA from colonies grown in 5-FOA and G418, cleaved it with *Taq*I or *Rsa*I, and recircularized the resulting fragments through ligation in dilute solution. We carried out inverse PCR to amplify fragments containing the end of the PiggyBac transposon and sequenced the PCR product (see *Materials and Methods*).

For wild-type Ste12, we obtained a total of 327 significant insertion peaks throughout the genome (Figure S2A). A 7-mer analysis of the 100-bp region around each insertion found that highly enriched 7-mers include the common sequence TGAAAC (Figure 1C), indicating that the Ste12-PBase fusion protein most frequently deposited the transposon near canonical Ste12-binding sites, as previously shown with the calling-card method (Wang *et al.* 2007). Nearly one-half (49.2%) of the insertions had such a canonical site within 200 bp (Figure 1D), which is comparable to the detection resolution from the chromatin immunoprecipitation sequencing method (Zheng *et al.* 2010). By assigning the insertion peaks to nearby genes (see *Materials and Methods*), we obtained a total of 264 gene targets. Gene ontology (GO) analysis revealed that these genes are most enriched [false discovery rate (FDR) < 0.001] for cell fusion or pheromone response pathways (Figure S2B). Thus, while Ste12-PBase was expressed under the control of the *GAL1* promoter, which is much stronger than the *STE12* promoter, the insertions fell into expected genes, suggesting that the high expression did not substantially affect Ste12 binding to the yeast genome.

The genome-wide insertion patterns for the six Ste12 variants were also characterized by the calling-card method (Figure 1E). Individual Ste12 variants showed a high degree of overlap between their experimental replicates [Figure 1, F and G; Pearson correlation coefficient ranged from *r* = 0.69–0.91, apart from A160P (*r* = 0.37), which resulted in the detection of only a few calling-card insertion peaks and thus had a relatively low correlation coefficient]. The fact that the variants yielded reproducible data also suggests that the high Ste12-PBase expression did not lead to adventitious nonspecific binding. However, pairwise comparisons revealed that the overlap among variants ranged only between 10 and 20%. Even for the two variants with the highest degree of overlap in target sites, K146D and K152L, 80% of the sites differed, despite their similar organismal phenotypes (Figure S3A). For those sites that were common in all the variants, we found that their nearby genes are classical targets of Ste12, and critical to either the mating or invasion phenotype (*e.g.*, *KAR4*, *FUS1*, and *TEC1*; Figure S3B). These results indicate that single-amino acid changes in the Ste12 DNA-binding domain led to many gains and losses of binding sites throughout the genome. However, even though the Ste12 variants bound overall to highly divergent sets of genomic sites, it is the binding sites that are in common that likely explain the key phenotypic differences.

### Transcriptome profiling of wild-type and variant Ste12 proteins

**Figure 2:**
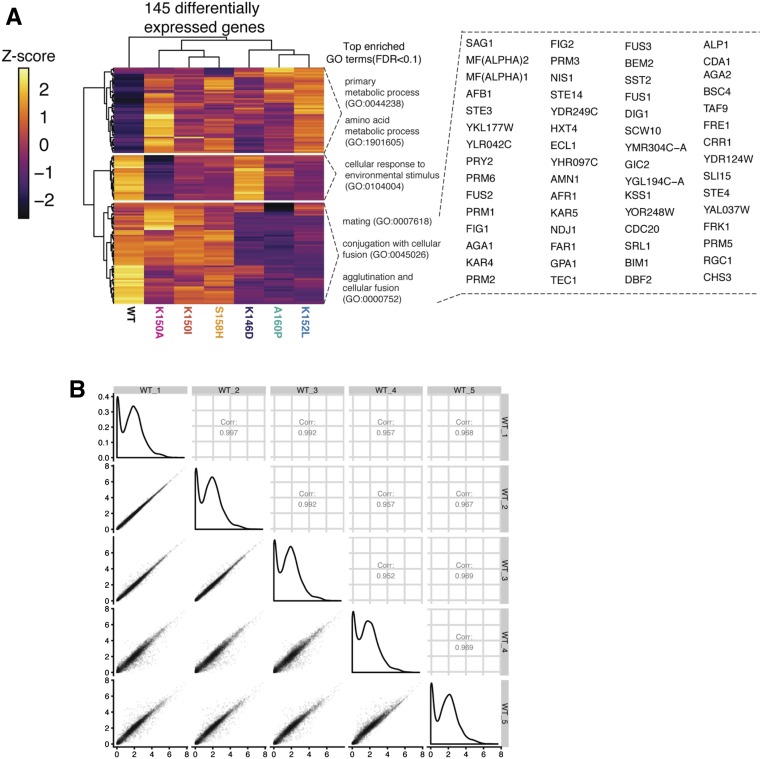
RNA-seq reveals gene targets of Ste12 and six variants. (A) Heatmap showing the clusters of differentially expressed genes across variants. The colors in the heatmap indicate the expression level of each gene normalized by library size, log-transformed and then mapped to the z-score. The top enriched GO terms in each cluster are included. The differentially expressed genes of the bottom cluster are shown on the right. (B) Correlation analysis between the replicates of WT Ste12 in the RNA-seq experiments. Left of the diagonal: correlation plots showing the Pearson’s correlations (*r*) between pairs of the replicates of WT Ste12 in the RNA-seq experiments. Diagonal: plots showing the distribution of the transcriptome of each replicate of the WT Ste12. Right of the diagonal: correlation plots showing the Pearson’s correlations (*r*) of the transcriptome of each pair of replicates of the WT Ste12. FDR, false discovery rate; GO, gene ontology; RNA-seq, RNA sequencing; WT, wild-type.

We sought to compare the DNA-binding sites of the Ste12 variants with genome-wide expression patterns induced by these variant proteins. We generated variant Ste12 proteins by introducing single-amino acid changes into the *STE12* gene under the control of its own promoter and carried on a centromere-based plasmid, and transformed the plasmids into a *ste12*Δ version of the BY4705 strain, which was generated through a site-specific genomic deletion method (see *Materials and Methods*). Cells were grown to exponential phase and total RNA was obtained. We carried out RNA-seq (five replicates) to generate the transcriptome for each variant, which yielded a combined total of 145 differentially expressed genes from the six variant strains when compared with wild-type Ste12, with an FDR of 5%. Replicability of the assay was high (Figure 2B; Pearson correlation coefficient range *r* = 0.97–0.99). The numbers of differentially expressed genes in the variant strains ranged from 15 to 84, but the numbers of these genes did not correlate with the severity of the altered mating or invasion phenotypes.

Unsupervised hierarchical cluster analysis of the data revealed three main differentially expressed groups of genes (Figure 2A). We used GO annotations to identify significant biological process terms (FDR < 0.1) for each cluster. For the largest cluster (bottom, Figure 2A), the top enriched GO terms included mating, conjugation with cellular fusion, and agglutination. For the other two clusters, the top enriched GO terms were associated with primary metabolic process and cellular response to environmental stimulus. Thus, the examination of differential gene expression revealed major clusters of genes, with many of these concordant with changes in mating and invasion phenotypes.

### Loss of mating proficiency correlates with loss of DNA binding

**Figure 3:**
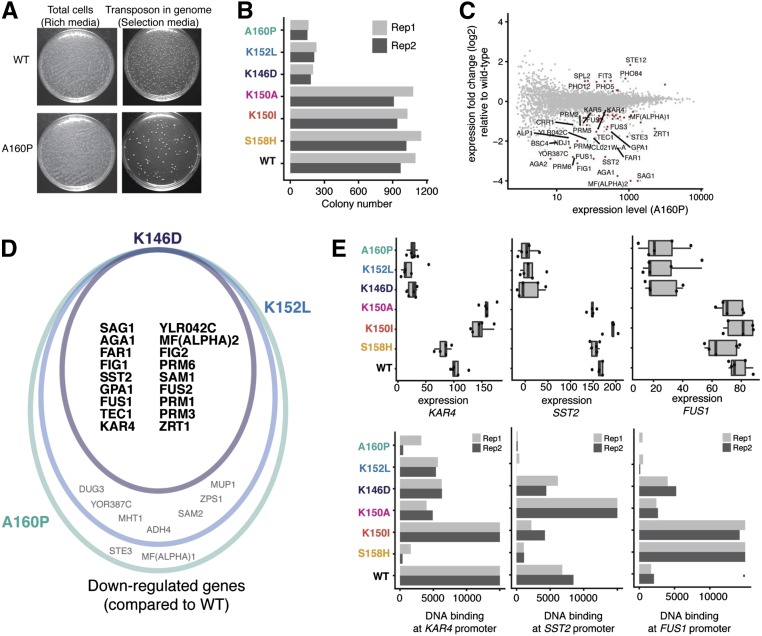
Variation in Ste12 DNA-binding domain drives difference in mating through altered binding. (A) Cell growth in rich medium and selection medium for WT Ste12 and the A160P variant. (B) Bar plot showing colony numbers after selection across all variants. Variants with different phenotypes are annotated by different colors. (C) Plot showing differentially expressed genes between the A160P variant and WT Ste12; the *x*-axis shows the total expression level for each gene in A160P while the *y*-axis shows the expression fold change relative to WT Ste12. Red color labels differentially expressed genes and the genes with log_2_ (fold change) > 1 are labeled with names. (D) Venn diagram showing overlap among downregulated genes for the mating-deficient variants (K146D in purple, A160P in green, and K152L in blue). (E) Top: box plots showing gene expression (fragments per million) among all variants for the genes *KAR4*, *SST2*, and *FUS1*. Lower: bar plots showing DNA binding (represented as fragments per million of total unique insertion events) among all variants for the genes *KAR4*, *SST2*, and *FUS1*. Rep, replicate; WT, wild-type.

Amino acid changes in the DNA-binding domain of a transcription factor potentially disrupt its DNA-binding affinity, resulting in the downregulation of common gene targets and phenotypic changes. To determine whether substitutions in Ste12 reduce its binding to genomic DNA, we compared DNA-binding activities across variants as the number of G418-resistant colonies (transposition efficiency); interaction between Ste12 and DNA provides the basis for the acquisition of the *KanMX* marker and G418 resistance. We found that the transposition efficiencies of the three mating-defective variants were only ∼10% of the wild-type and mating-competent variants (Figure 3, A and B), suggesting that the mating-defective variants have decreased affinities for DNA. For example, A160P, a mating-deficient variant, resulted in the fewest insertion sites in the calling-card experiment (Figure 3A), and the vast majority of genes differentially expressed between A160P and wild-type were downregulated in A160P (50 out of 63, Figure 3C). Eighteen out of 50 of the significantly downregulated differentially expressed genes in the A160P variant were also expressed at a lower level in the other mating-deficient variants (Figure 3D), and these overlapping downregulated genes act at every step of mating process. For example, *GPA1* encodes the α subunit of the G protein that mediates pheromone sensing at the initial step of the process. *FAR1* functions at an early step by mediating cell cycle arrest and stimulating the polarized growth of the cell toward its mating partner. *AGA1*, *SAG1*, and F*IG*2 act at a later step of cell agglutination, contributing to cell–cell contact and the generation of an ultrastructure favorable for zygote formation. *FUS1*, *FUS2*, and *KAR4* act at the terminal stage of the mating process of cell fusion and nuclear fusion/karyogamy formation. Fourteen of 18 (∼78%) of the overlapping downregulated genes were found to be direct targets of the wild-type Ste12 based on the genome-binding profile. Of the 14, four genes (*KAR4*, *SST2*, *PRM1*, and *PRM3*) had a reduction in the DNA-binding signal (*P*-value < 0.05; proportion test) in all mating-deficient variants, and 10 genes (*P*-value < 0.05; proportion test) in at least one mating-deficient variant (Figure 3E).

In summary, we conclude that amino acid changes in the DNA-binding domain of Ste12 that disrupt its interaction with the genome can be distinguished by the many fewer calling-card insertions that were detected. This disruption results in loss-of-binding to critical gene targets and markedly reduces the expression of mating-related genes, which would be expected to dramatically decrease the mating proficiency of the mating-deficient variants (K146D, K152L, and A160P).

### Altered DNA binding and transcriptomes in hyper-invasive variants

**Figure 4:**
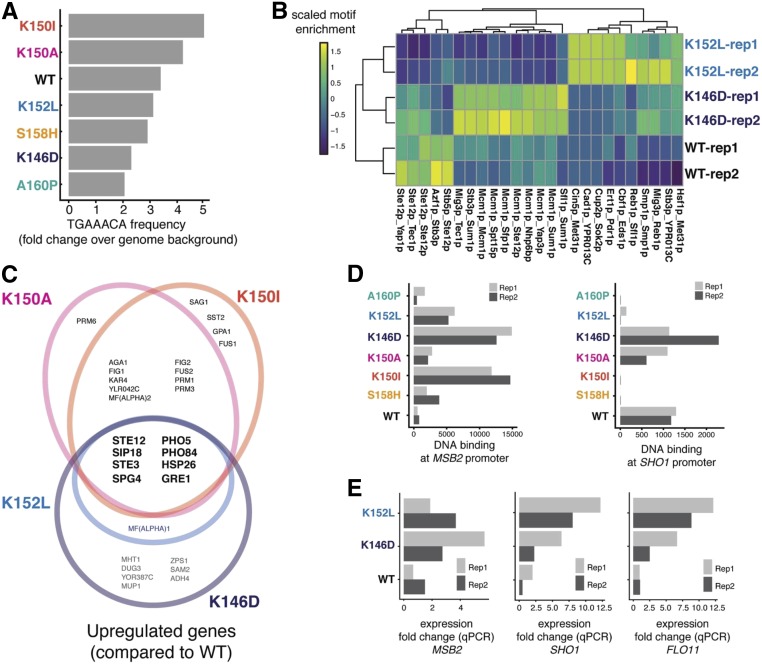
Patterns of binding and activation in invasion-deficient and hyper-invasive Ste12 variants. (A) Bar plots showing enrichment of TGAAACA frequency relative to the genome background for the Ste12 variants. (B) Heatmap showing enrichment of motif pairs found near binding sites for WT Ste12, and the K146D and K152L variants. (C) Venn diagram showing overlap in upregulated genes among hyper-invasive variants (K146D is in purple, K152L is in blue, K150A is in pink, and K150I in orange) (D) Bar plot showing the DNA-binding (represented as fragments per million of total unique insertion events) among all variants for the genes *MSB2* and *SHO1*. (E) Bar plots showing qPCR expression fold change (over WT) for genes *MSB2*, *SHO1*, and *FLO11*. qPCR, quantitative PCR; Rep, replicate; WT, wild-type.

Two of the mating-defective variants (K146D and K152L) invade as well as or better than the wild-type, indicating that they are not fully defective. One possibility is that they maintain interactions with some cofactors while losing interactions with others or directly with DNA, resulting in distinct variant-specific binding and expression patterns. To test this possibility, we used the genome sequences near Ste12-PBase insertion peaks and examined the frequencies of Ste12 canonical sites across the variants. We found that the K152L and K146D variants showed a reduction in binding relative to wild-type Ste12 at canonical sites required for mating (Figure 4A). To capture interactions with cofactors, we calculated the frequencies of transcription factor motif pairs present at sites bound by the wild type, and the K146D and K152L variants. Pairs containing the well-characterized Mcm1-binding site (Hwang-Shum *et al.* 1991) were significantly enriched in K146D compared to the wild type, suggesting that interaction with Mcm1 may be implicated in altered Ste12 binding (Figure 4B). The K146D variant may preferentially bind to promoter regions containing a site for Mcm1, a protein that can induce invasive growth (Birkaya *et al.* 2009), to contribute to its unique genome-binding patterns and hyper-invasive phenotype. However, the presence of cofactor motifs at bound sites was not sufficient to explain the function of hyper-invasive variants other than K146D.

We integrated both genome-binding and transcriptome profiles in an effort to uncover the mechanism underlying the altered invasion phenotype seen in the hyper-invasive variants (K146D, K152L, K150I, and K150A). Transcriptome analysis revealed that shared upregulated genes in hyper-invasive variants are associated with the cellular response to osmotic stress (*e.g.*, *SIP18*, *SPG4*, and *GRE1*; Figure 4C). When yeast cells are exposed to a hyper-osmotic condition, osmotic stress responses are triggered, and cells form long projections and become hyper-invasive due to Kss1, a key MAP kinase in the invasion pathway (Davenport *et al.* 1999). The signaling events that lead to osmotic stress responses depend on the membrane environmental sensors Sho1 and Msb2 (Tatebayashi *et al.* 2006). Sho1 is one of the G protein-coupled receptors that act as transmembrane osmosensors (Posas and Saito 1997) and Msb2 is a membrane mucin protein (Tatebayashi *et al.* 2007). Based on the genome-binding profile data, the *SHO1* and *MSB2* genes were direct targets of wild-type Ste12, suggesting a potential role for Ste12 upstream of the osmotic stress response pathway.

For the hyper-invasive variants with reduced mating (K146D and K152L), while they failed to efficiently bind to the Ste12 consensus site, resulting in downregulation of critical mating genes, both variants had an increased insertion signal (*P*-value < 0.05; proportion test) for *MSB2* and K146D had a higher insertion signal for *SHO1* (although not at a significant level) (Figure 4D). Due to their low levels of transcription, the *SHO1* and *MSB2* genes were not different from wild-type in the full transcriptome analysis. However, we detected higher *MSB2* and *SHO1* expression in K146D and K152L variants than wild-type using RT-PCR, as well as higher expression of *FLO11*, a final target in the Sho1-sensing pathway (Figure 4E). In the promoter regions of *SHO1* and *MSB2*, an Mcm1 site is found close to transposon insertion sites of K146D, suggesting that this cofactor interaction may partially underlie the hyper-invasive phenotype.

Distinct from these two mating-defective variants, the mating-competent and hyper-invasive K150A and K150I variants were the most enriched for the canonical Ste12-binding site, showing greater enrichment for this site than even the wild-type Ste12 (Figure 4A and Figure S4). This result indicates that these two variants are intact for their DNA-binding activity and bind to the canonical site like the wild-type does, consistent with their mating-competent phenotypes.

Overall, by integrating both transcriptome and genome-binding profiles, we found a small, related subset of genes involved in the osmotic stress response that appear critical for the altered invasion phenotype seen in the hyper-invasive variants. These results also suggest the possibility of at least three mechanistic bases that contribute to variant-specific binding events and gene expression: one that uses canonical Ste12 sites (K150A and K150I), one that does not (K152L), and one that may involve cofactor interactions (K146D).

### Binding patterns of Ste12 variants *in vitro*

Because the binding patterns of the Ste12 variants *in vivo* can be influenced by cofactor interactions that affect the sequence contacted by Ste12, we asked whether direct binding specificity *in vitro* was altered. To characterize the *in vitro* DNA-binding preferences of the variants, we used the HT-SELEX method (Jolma *et al.* 2015). In our use of this method, purified Ste12 protein was incubated with a large and random (N36) pool of DNA fragments. The DNA fragments bound to protein were isolated, amplified by PCR, and incubated again with protein through five rounds, with the PCR product from each round used for high-throughput DNA sequencing. Motif analysis of the SELEX data for wild-type Ste12 shows that the canonical binding site was enriched over the rounds of selection, and WebLogos of the most significant motifs for each variant show patterns similar to wild-type Ste12 (Figure S6). K146D and K152L were exceptions, showing either no enriched motif (K146D) or a weakly significant, degenerate version of the canonical Ste12 site (K152L) (Figure S6).

**Figure 5:**
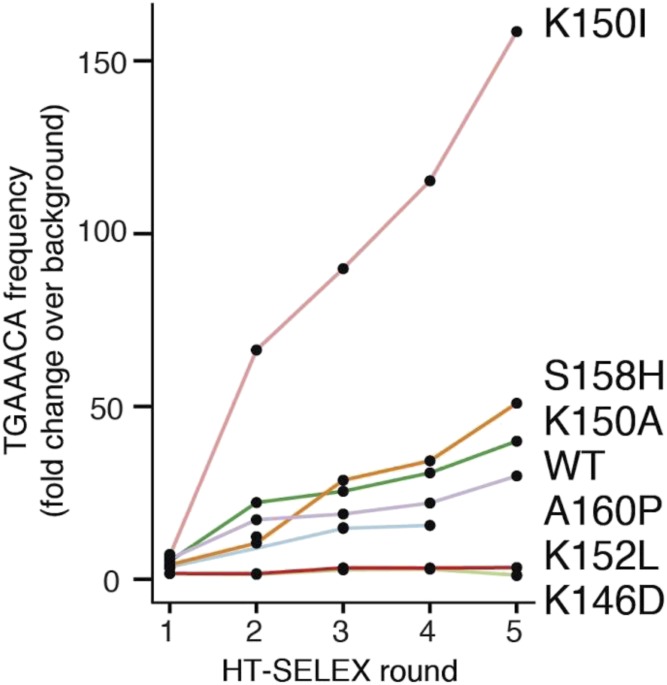
Line plot showing TGAAACA frequency in HT-SELEX for WT Ste12 and six variants. The *x*-axis shows consecutive rounds of HT-SELEX binding assays. The *y*-axis shows the enrichment for the TGAAACA 7-mer over the random input sequence background for each variant. WT, wild-type. HT-SELEX, high throughput systematic evolution of ligands by exponential enrichment.

The TGAAACA sequence was preferred for the K150I and K150A variants, but not for the mating-deficient variants, especially K152L and K146D, which showed no enrichment (Figure 5A). The K150I variant showed greater enrichment *in vitro* for the TGAAACA sequence than wild-type, and also showed a higher enrichment than wild-type for this sequence in its *in vivo* genome-binding pattern. The *in vitro* binding preferences of Ste12 variants for the canonical Ste12-binding site generally correlated with those preferences *in vivo*. Furthermore, no Ste12 variants showed novel binding specificity *in vitro*, suggesting that their divergent binding patterns *in vivo* are driven by altered affinity for the canonical Ste12 site and the presence of binding cofactors rather than changes in sequence specificity.

## Discussion

The common assumption that transcriptional regulation is orderly, and hence predictable, has been shaped by the conservation of gene expression patterns across species, conserved and modular transcription factor domains, and a long history of experiments using simpler prokaryotic systems and model regulatory regions in eukaryotes. Our results add to the emerging argument that transcriptional regulation can be readily rewired, changing the underlying transcriptional circuits in various ways while preserving phenotypic outputs. In evolution, this extensive rewiring can occur even among closely related species, and is facilitated by the rapid gain and loss of short *cis*-regulatory sequences, or by variation in transcription factors (Dalal and Johnson 2017).

The *S. cerevisiae* Ste12 protein regulates the traits of mating and invasion by interacting with other transcription factors, to bind and activate distinct sets of genes in response to mating pheromone or nutrients, respectively. Single-amino acid substitutions in the Ste12 DNA-binding domain can result in dramatically altered phenotypes, such as a shift in preference toward either mating or invasion, or a hyper-invasive phenotype that is independent of the invasion cofactor Tec1. We demonstrate here that these variant Ste12 proteins lead to extensive changes in genome-wide binding patterns and transcriptional outputs.

Although individual Ste12 variants showed highly reproducible binding events in the calling-card assay, there was little overlap in binding among variants, even for those with similar mating and invasion phenotypes. This lack of overlap indicates that the subtle changes in the Ste12 protein led to numerous gains and losses of individual binding sites throughout the genome. Nevertheless, it is difficult to pin down the mechanistic basis for these gains and losses of sites without additional experimentation. In three cases (K146D, K152L, and A160P), the mating deficiency caused by a variant could be explained by a marked reduction in DNA-binding affinity, based on many fewer calling-card insertions and either a failure to enrich or reduced enrichment of the consensus site in the SELEX experiment. This failure to efficiently bind to Ste12 consensus sites led to the decreased expression of a small set of common genes, several of which function in the mating process. However, that these variants maintain, or even increase, their invasiveness suggests that they are not completely defective proteins and that separation-of-function alleles can arise readily. Moreover, the maintenance of invasion function in variants defective for DNA binding implicates cofactors in the new binding and expression patterns. Variant proteins may maintain interactions with some cofactors while losing interactions with others, resulting in distinct variant-specific expression patterns. Further analyses of DNA binding by these variants could use approaches such as gel mobility shift assays to obtain quantitative affinity data. In addition, carrying out these assays on a series of promoters from pheromone-responsive or invasion-specific genes in the presence of purified cofactors such as Tec1, Mcm1, and Matα1 might reveal protein interactions that are either enhanced or reduced by the Ste12 substitutions.

The three variants that resulted in near wild-type mating (K150A, K150I, and S158H) showed similar numbers of calling-card insertions and similar enrichment of the consensus site in the SELEX experiment as the wild-type protein. These results imply that DNA-binding affinity is likely to be intact in these variants. Variants that bind to the consensus site like wild-type but result in a new phenotype also suggest a change in the interaction with cofactors. The K150A and K150I variants lead to a hyper-invasive phenotype, and these two uniquely increased the expression of genes responsive to osmotic stress. The hyper-invasive variants that are mating-defective (K146D and K152L) increased the expression of additional genes, indicating that multiple paths to a hyper-invasive phenotype are possible.

Although the Ste12 transcription factor variants easily gained or lost binding sites, most variant-specific binding events seemed to have little to no effect on the organismal phenotypes of mating and invasion. We attribute this phenotypic robustness to the underlying regulatory network architecture, which will amplify the effects of specific binding events and expression through feedback loops, motif degeneracy, and binding site redundancy while canceling the effect of aberrant binding and expression events. At the same time, the ease with which a single-amino acid substitution in Ste12 shifts phenotype suggests that a few novel binding events and expression changes suffice as starting points for the rewiring of regulatory programs in evolution (Dalal and Johnson 2017).

A major goal in functional genomics is the prediction of variant effects from sequence alone. However, current variant effect prediction algorithms and computational structure-based approaches (Barrera *et al.* 2016) provide minimal annotations, such as loss of DNA-binding or likely pathogenesis in humans. In the case of Ste12 variation, it was not possible to attribute specific phenotypes of mating or invasion based solely on the identity of the single-amino acid changes in the Ste12 protein sequence. This failure to predict the *in vivo* effects of transcription factor variants calls for the systematic large-scale functional interrogation of human transcription factor variants *in vivo*. Thus far, there are few options to conduct such studies at sufficient scale in human cells. In this regard, it is notable that we detected genome-wide binding patterns of Ste12 variants in the presence of the wild-type protein. Expressing libraries of transcription factor variants from a common genomic site in human cells containing their respective wild-type proteins is far more feasible than the prospect of engineering many hundreds of endogenous loci. While most transcription factor variants are unlikely to contribute to phenotypic changes, identifying those with major downstream effects remains a critical challenge.
